# Measurement of trihydroxy-linoleic acids in stratum corneum by tape-stripping: Possible biomarker of barrier function in atopic dermatitis

**DOI:** 10.1371/journal.pone.0210013

**Published:** 2019-01-04

**Authors:** Takahito Chiba, Takeshi Nakahara, Futoshi Kohda, Toshio Ichiki, Motomu Manabe, Masutaka Furue

**Affiliations:** 1 Department of Dermatology and Plastic Surgery, Akita University Graduate School of Medicine, Akita, Japan; 2 Department of Dermatology, Graduate School of Medical Sciences, Kyushu University, Fukuoka, Japan; 3 Department of Dermatology, Aso Iizuka Hospital, Fukuoka, Japan; INSERM, FRANCE

## Abstract

Epidermal ceramides are indispensable lipids that maintain the functions of the stratum corneum. Esterified omega-hydroxyacyl-sphingosine (EOS) ceramide with a linoleate moiety is one of the most important ceramide species for forming cornified lipid envelopes. This linoleate moiety is eventually metabolized to trihydroxy-linoleic acid (triol, 9,10,13-trihydroxy-11*E*-octadecenoic acid). Thus, we assumed that a decrease of triols might reflect skin barrier dysfunction. Against this background, the purposes of this study were to measure the triols by a simple tape-stripping method and to determine the correlation between the amount of triols and transepidermal water loss (TEWL) as an indicator of barrier dysfunction in atopic dermatitis patients. Twenty Japanese subjects with normal skin and 20 atopic dermatitis patients were enrolled in this study. TEWL was measured and triols of the stratum corneum were analyzed by tape-stripping. The results showed for the first time that triols in the stratum corneum could be simply measured using the tape-stripping method. The triol levels in atopic dermatitis patients were much higher than those in healthy subjects. Moreover, the triol levels correlated with TEWL of non-lesional forearm skin in patients with atopic dermatitis. The results suggest that the assaying of triol levels via non-invasive tape-stripping could be beneficial for monitoring barrier function in atopic dermatitis.

## Introduction

**Fig 1:**
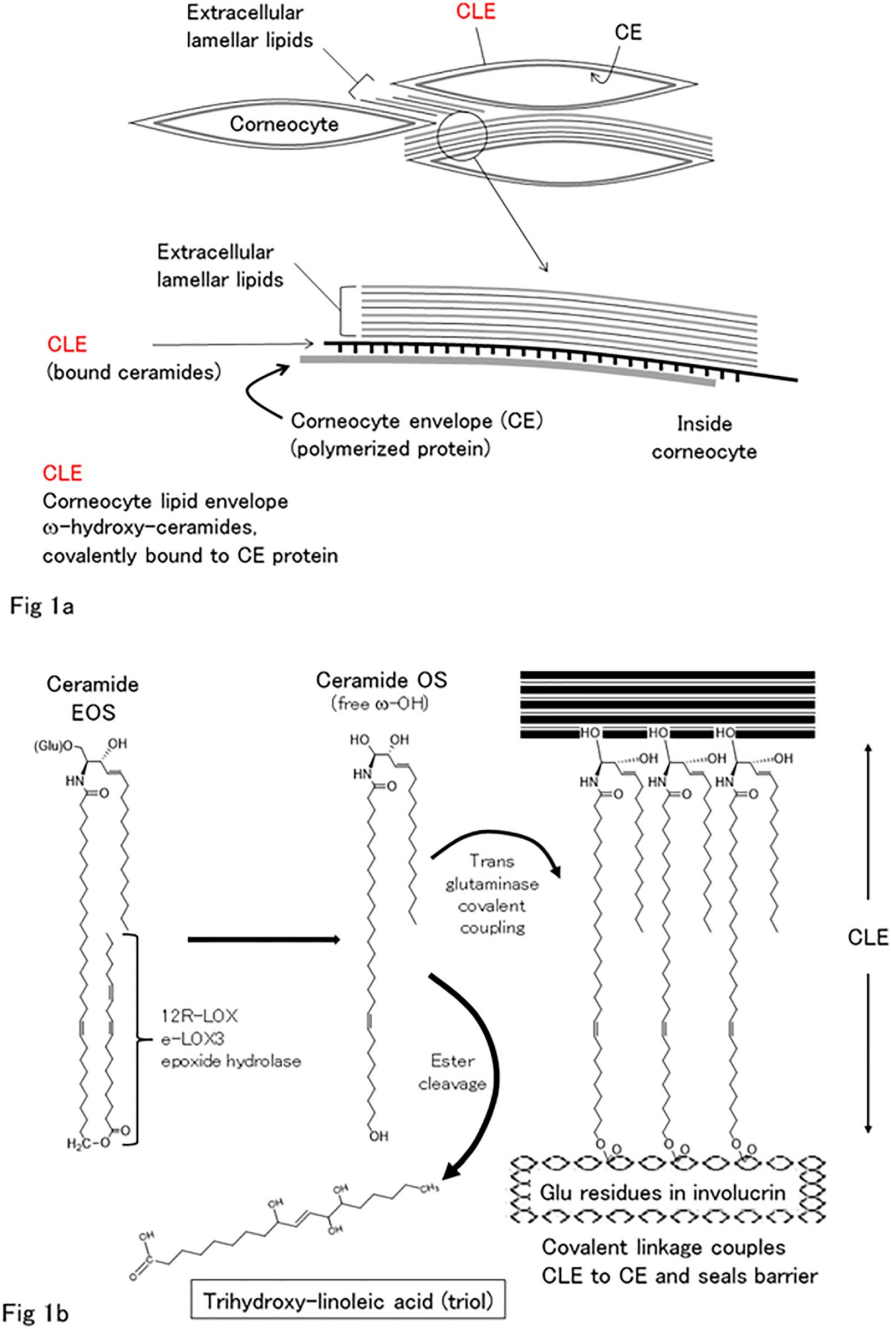
Structures of stratum corneum and formation of the corneocyte lipid envelope (CLE). (a) An image of the cornified barrier obtained by electron microscopy [5]. In stratum corneum, the corneocytes are bound together by the fusion of three substructures: (i) polymerized proteins forming the corneocyte envelope (CE), (ii) extracellular lamellar lipids between cells, and (iii) a monolayer of covalently bound ceramides and fatty acids, the corneocyte lipid envelope (CLE), covering the CE and forming a scaffold for the extracellular lamellar lipids. (b) Our working hypothesis [9] requires the LOX-catalyzed oxidation of the linoleate in esterified omega-hydroxyacyl-sphingosine (EOS), ceramide, facilitating hydrolysis of the ester bond, separating ceramide OS for coupling to the CE protein by transglutaminase, finally forming the CLE. Trihydroxy-linoleic acid (triol) cleaved from ceramide EOS is produced as the final metabolite. (i) 9-hydroperoxy-11,12-octadecadienoate, (ii) 9,10-epoxy-11*E*-13-hydroxyoctadecenoate (iii) 9,10,13-trihydroxy-11*E*-octadecenoate.

The barrier function of the skin is mainly provided by the stratum corneum. Although individual corneocytes in the stratum corneum act as a major structure forming a physical barrier, extracellular lamellar lipids (ECLLs) containing abundant ceramides also play an important role in the barrier structure [1]. These are often described as brick (corneocytes) and mortar (ECLLs) structures. There is also a single thin layer between corneocytes (crosslinked protein) and ECLLs, which can be observed using an electron microscope, called the corneocyte lipid envelope (CLE); this is considered to be an integral component for combining the corneocyte envelope with ECLLs [1, 2] (Fig 1a). Thus, insufficiency of CLE formation induces skin symptoms and increased transepidermal water loss (TEWL) since an appropriate stratum corneum cannot be constructed [3, 4].

To construct CLE, epidermal-specific ceramides to which linoleic acid is bound, glucosyl ω-O-acyl-ceramide and its deglucosylated product, are used [6, 7]. Esterified omega-hydroxyacyl-sphingosine (EOS) is the main epidermal ceramide in four types of ω-O-acyl-ceramide [8]. As a currently acceptable CLE-forming model, the linoleate moiety of ceramide EOS is first oxidized by 12*R*-lipoxygenase (12*R*-LOX) [1, 9] and then isomerized by epidermal lipoxygenase-3 (eLOX3) [1, 10]. After epoxide hydrolysis of the epoxyalcohol moiety by epoxide hydrolase-2 and -3 [11], ceramide EOS is further converted to ceramide omega-hydroxy-sphingosine (OS), which is covalently attached to the surface of the cornified envelope (CE) composed of cross-linked proteins [12–15] (Fig 1b). This model is also supported by the existence of a specific chirality of 9*R*,10*R*-*trans*-epoxy-13*R*-hydroxy-octadec-11*E*-enoate enantiomer (12*R*-LOX metabolite) and 9*R*-hydroxy-10*E*,12*Z*-octadecadienoic acid in human epidermis [9] [16]. This CLE formation concept is clinically supported by the fact that the mutation of epidermal-specific lipoxygenases such as 12*R*-LOX and eLOX3, which are involved in CLE formation, causes a barrier-related disease, autosomal recessive congenital ichthyosis (ARCI) [17, 18]. In atopic dermatitis, it was reported that ceramide OS decreases in the cornified layer [19], and the resulting paucity of CLE components exacerbates skin dryness or other symptoms [19–21]. These findings indicate that the amount of ceramide OS as a final product of ceramide EOS in stratum corneum influences skin barrier function. Therefore, it might be possible to evaluate skin barrier function or CLE construction by measuring ceramide OS. However, ceramide OS are difficult to measure using LC-MS because they have carbon chains of different lengths (at least C48–C54) [8, 22].

Considering a series of ceramide EOS metabolic processes, we focused on the final metabolite, trihydroxy-linoleic acid (9,10,13-trihydroxy-11*E*-octadecenoic acid), which is cleaved from ceramide EOS. As mentioned above, it is hypothesized that linoleate moiety in the epidermis could be catalyzed by three steps of 12*R*-LOX, eLOX3, and epoxide hydrolase and that the trihydroxy-hydrolysis products (trihydroxy-linoleic acid, triol) of these intermediates might be the final oxidized products of the pathway [9, 11, 16] (Fig 1b). We reported the existence of a level of trihydroxy-linoleic acid that was measurable using LC-MS in human and pig epidermis. This indicates that these measurable triols might indirectly reflect the amount of ceramide OS, which is a main component of CLE. Therefore, we sought to identify and measure trihydroxy-linoleic acid in the stratum corneum using a simple tape-stripping method and to determine its correlation with TEWL as an indicator of barrier dysfunction.

Accordingly, the objectives of the current study were: i) to develop a simple and practical method for measuring trihydroxy-linoleic acid using stripped tape specimens; ii) to compare the amount of trihydroxy-linoleic acid between healthy subjects and atopic dermatitis patients, with the latter representing CLE construction failure and skin barrier impairment; and iii) to determine the correlation of the amount of trihydroxy-linoleic acid with barrier function (TEWL).

## Materials and methods

### Participants

Twenty adult patients from Aso Iizuka Hospital (mean age 43 years; 9 females, 11 males) with atopic dermatitis diagnosed in accordance with the criteria of atopic dermatitis clinical guidelines from the American Academy of Dermatology [23] were registered with the study after their written informed consent had been obtained. Each patient was examined for IgE, peripheral eosinophil count, thymus and activation-regulated chemokine (TARC), lactate dehydrogenase (LDH), and SCORing Atopic Dermatitis (SCORAD). The patients’ detailed clinical and laboratory data are shown in S1 Table. The mean SCORAD in the atopic dermatitis patients was 36 (range 10–80). The correlation between SCORAD and the other laboratory data is shown in S1 Table. Before the trihydroxy-linoleic acid measurement, the patients were allowed to apply local steroids or calcineurin inhibitors to their eczematous lesions (except for the investigated non-eczematous lesions). Patients undergoing systemic immunosuppressive or anti-histamine therapy were included. Staff (mean age 42 years; 11 females, 9 males) at Aso Iizuka Hospital who did not have any skin diseases were also included as a healthy group, from whom informed consent was also obtained. The research was approved by the Ethics Committee of Aso Iizuka Hospital.

### Measurement of TEWL and stratum corneum stripping

TEWL measurements and stratum corneum stripping were performed in an air-conditioned room (temperature 24°C; humidity 40%). Each subject washed their forearm and forehead with makeup remover and a face wash, and was then allowed to acclimate to the conditions for 15 min. The locations at which measurements were performed were the center of the forehead and the outer forearm, avoiding sites of eczema in the case of the atopic dermatitis group. TEWL was measured twice with a closed-chamber VAPO SCAN AS-VT100RS (Asch Japan Co., Ltd., Tokyo, Japan) and the mean value was determined. Subsequently, stratum corneum specimens were collected from the same sites (forehead and forearm) by the tape-stripping method as described in previous reports [22, 24]. Briefly, adhesive tape (tape size, 6 × 2 cm; Skinergate Spatt tape; Nichiban, Tokyo, Japan) was placed on the above skin sites and subjected to a constant pressure. The tape obtained from the first attachment, which reflects the surface of the cornified layer, was used for measurement. Half of the obtained tape was used for trihydroxy-linoleic acid analysis and the other half for protein analysis. Until all specimens had been prepared, the tape samples were stored at −30°C.

### Analysis of trihydroxy-linoleic acid

Half of the tape (2 × 3 cm) was immersed in methanol (Kanto Chemical Co., Inc., Tokyo, Japan) and sonicated for 5 min. The extracts were dried using a nitrogen stream and then dissolved in 200 μl of methanol again. This lipid solution or authentic solution was applied to the following LC-MS method. Trihydroxy-linoleic acid (9,10,13-trihydroxy-11*E*-octadecenoic acid) is considered to include a total of eight isomers (S1 Fig); a mixture of these eight authentic trihydroxy-linoleic acids, triols-1 to 8, was kindly provided by Dr. Alan R. Brash (Vanderbilt University School of Medicine, Nashville, TN). A Shimadzu 8050 triple-stage quadrupole mass spectrometer equipped with an ESI ion source (Shimadzu, Kyoto, Japan) and a Shimadzu HPLC system [Nexera X2 LC0AD; CBM-20A (system controller), LC-30AD (solvent delivery unit), SIL-30AC MP (autosampler), CTO-20AC (column oven)] were employed. The column was a CAPCELL PAK C18 type IF (50 × 2.0 mm I.D., 2.0 μm; Shiseido, Tokyo, Japan) and was used at 40°C. The mobile phase consisting of 10 mM ammonium acetate (solvent A) and methanol (solvent B) was used with a gradient elution of A:B = 85:15 to 0:100 (0–5 min) at a flow rate of 0.3 ml/min. ESI-MS conditions were as follows: spray voltage, −3000 V; spray gas, 2 L/min, nitrogen; collision gas, argon; heating gas, 10 L/min; heating block temperature, 120°C; drying gas, 10 L/min; ion source temperature, 400°C; and ion polarity, negative. The amount of trihydroxy-linoleic acid was calculated from the concentration-adjusted authentic standard reagent (triol-1).

### Protein analysis

Soluble proteins were obtained from the other halves of the tapes by immersing them in 0.1 M NaOH 1% (w/v) sodium dodecyl sulfate (Wako, Osaka, Japan) aqueous solution and then incubating them at 60°C for 2 h. After this incubation, the solution was neutralized with 1 M HCl (Wako). The level of soluble protein in each sample solution was measured using a BCA kit (Thermo Scientific, Rockford, IL, USA) along with bovine serum albumin as a protein standard [25]. Concentrations of trihydroxy-linoleic acid in both groups were corrected by the total protein content.

### Real-time reverse-transcription (RT)-PCR analysis

Primary normal human epidermal keratinocytes (NHEK) were obtained from Clonetics-BioWhittaker (San Diego, CA, USA). These cells were grown in culture dishes at 37 °C and 5% CO_2_ and cultured in serum-free keratinocyte growth medium (KURABO, Osaka, Japan) supplemented with bovine pituitary extract, recombinant epidermal growth factor, insulin, hydrocortisone, and gentamycin. Culture medium was replaced every 2 days. Near confluence (70%–90%), cells were disaggregated with 0.25% trypsin/0.01% ethylenediaminetetraacetic acid and subcultured. Second-to-fourth-passage NHEK cells were used in all experiments. NHEK cells were stimulated for 24h with respective designated concentration of Interleukin-4, IL-4 (PeproTech, Rocky Hill, NJ, USA). 50The cells were then washed with phosphate-buffered saline (PBS) and total RNA was extracted using the RNeasy Mini kit (Qiagen, Courtaboeuf, France). RT was performed using a PrimeScript RT-reagent kit (Takara Bio, Otsu, Japan). Real-time RT-PCR was performed on the Mx3000p real-time system (Stratagene, La Jolla, CA, USA) using SYBR Premix Ex Taq (Takara Bio). Amplification was started at 95°C for 30 s as the first step, followed by 40 cycles of real-time RT-PCR at 95°C for 5 s and 60°C for 20 s. The amount of mRNA was normalized to that of β-actin using the ΔCt method, as described in the manufacturer’s protocol. The sequences of primers from Takara Bio and SA Biosciences (Frederick, MD, USA) are shown in S2 Table.

### Statistical analysis

Data are presented as mean and standard deviation (SD). Student’s *t*-test was used to determine the significance of differences between groups. Correlations among the clinical and laboratory data in atopic dermatitis patients were assessed by calculating the Pearson product-moment correlation coefficient.

## Results

### LC-MS analysis of trihydroxy-linoleic acid

**Fig 2:**
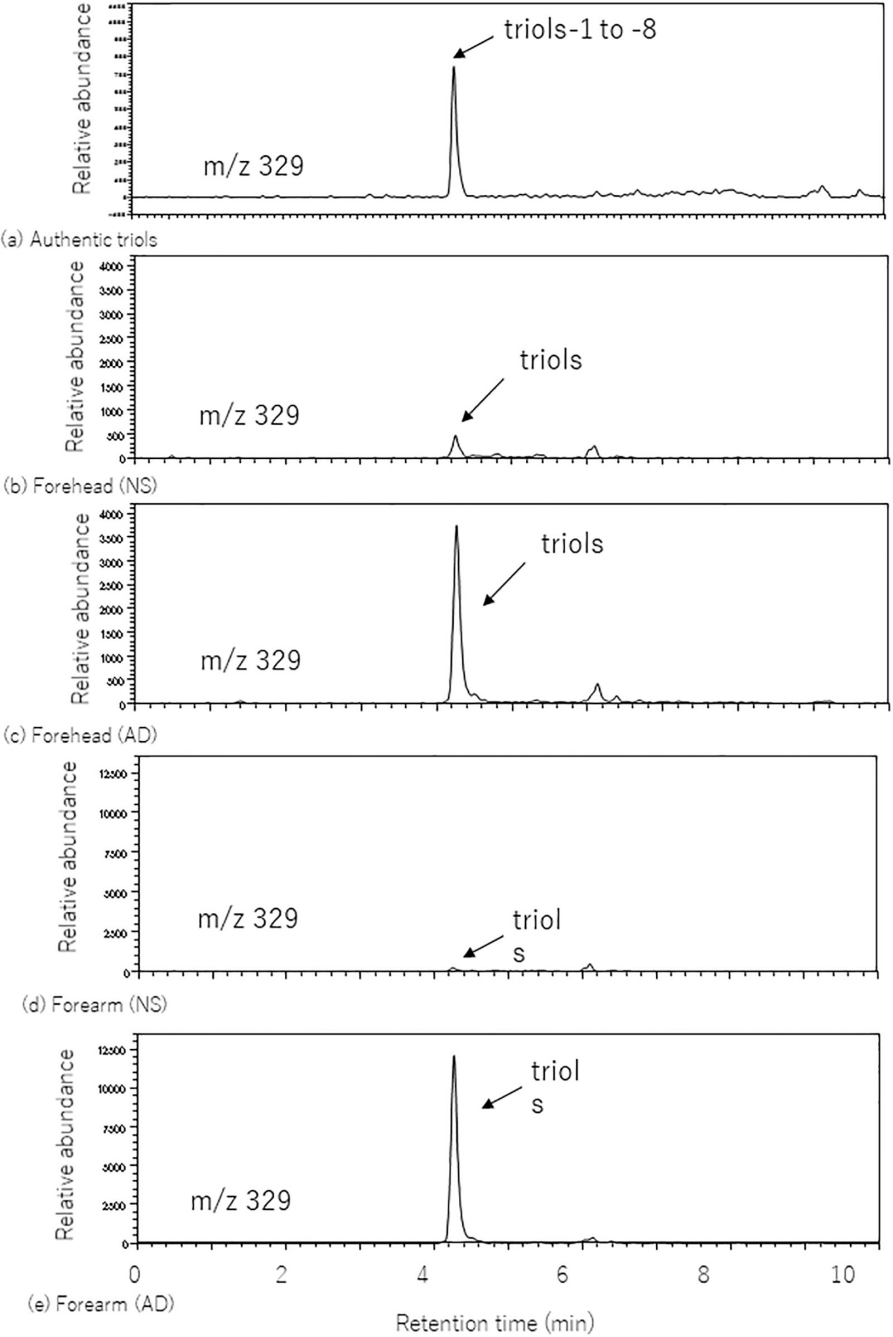
LC-MS analysis of triols. SIM chromatograms (m/z 329) of triols analyzed by reverse-phase LC-ESI-MS. (a) Analysis of a mixture of authentic triols-1 to -8 (2 ng) as a standard, (b) triols of forehead skin in normal subjects and (c) atopic dermatitis patients, and (d) triols of forearm skin in normal subjects and (e) atopic dermatitis patients.

LC-MS analysis of authentic trihydroxy-linoleic acids (mixture of triols-1 to -8) revealed a particularly prominent peak at ~4.2 min (Fig 2a), which was considered to represent all eight triols. The tape-stripping specimens from forehead or forearm in healthy subjects had the same retention time regarding the triol standards (Fig 2b and 2d). This single peak should be consistent with the set of eight enantiomers of trihydroxy-linoleic acid in human epidermis. The tape-stripping specimens from patients with atopic dermatitis also showed the same chromatogram (Fig 2c and 2e).

### Comparison between normal subjects and atopic dermatitis patients

As shown in Table 1, the mean TEWL in normal subjects was 32.9 ± 20.0 g/m^2^h in forehead and 12.6 ± 6.9 g/m^2^h in forearm. In contrast, the mean values for non-lesional skin in atopic dermatitis patients were 49.2 ± 35.5 g/m^2^h in forehead and 20.6 ± 13.4 g/m^2^h in forearm; this is indicative of the impairment of skin barrier function, as also described in a previous report [26, 27]. SCORAD also correlated with serum markers of atopic dermatitis, however, the values of TEWL and SCORAD were not necessarily correlated in our study (S3 Table). The mean concentrations of total trihydroxy-linoleic acids in normal subjects were 0.75 ± 0.74 pg/cm^2^ in forehead skin and 0.26 ± 0.16 pg/cm^2^ in forearm skin. In the atopic dermatitis group, the corresponding values were 2.08 ± 3.43 and 1.03 ± 2.28 pg/cm^2^, respectively. Next, we corrected the trihydroxy-linoleic acid amount with the protein amount of corneum. The mean concentrations of total trihydroxy-linoleic acids in normal subjects were calculated as 6 ± 5 pg/μg protein in forehead skin and 4 ± 4 pg/μg protein in forearm skin. In the atopic dermatitis group, the corresponding values were 15 ± 22 and 37 ± 58 pg/μg protein, respectively.

**Table 1:** Baseline characteristics and comparison of TEWL and the quantity of trihydroxy-linoleic acids between normal subjects and atopic dermatitis patients.

Variable	Normal subjects	Atopic dermatitis	*p* value
Number	20	20	-
Sex (female)	11	9	0.527
Age (years, mean±SD)	43.1±14.0	42.6±13.8	0.774
TEWL forehead (g/m^2^h, mean±SD)	32.9±20.0	49.2±35.5	0.01
TEWL forearm (g/m^2^h, mean±SD)	12.6±6.9	20.6±13.4	0.005
Trihydroxy-LA forehead (pg/cm^2^, mean±SD)	0.75±0.74	2.08±3.43	0.23
Trihydroxy-LA forearm (pg/cm^2^, mean±SD)	0.26±0.16	1.03±2.28	0.03
Trihydroxy-LA forehead (pg/μg protein, mean±SD)	6±5	15±22	<0.001
Trihydroxy-LA forearm (pg/μg protein, mean±SD)	4±4	37±58	<0.001

### Correlation of TEWL and trihydroxy-linoleic acids in atopic dermatitis patients

**Fig 3:**
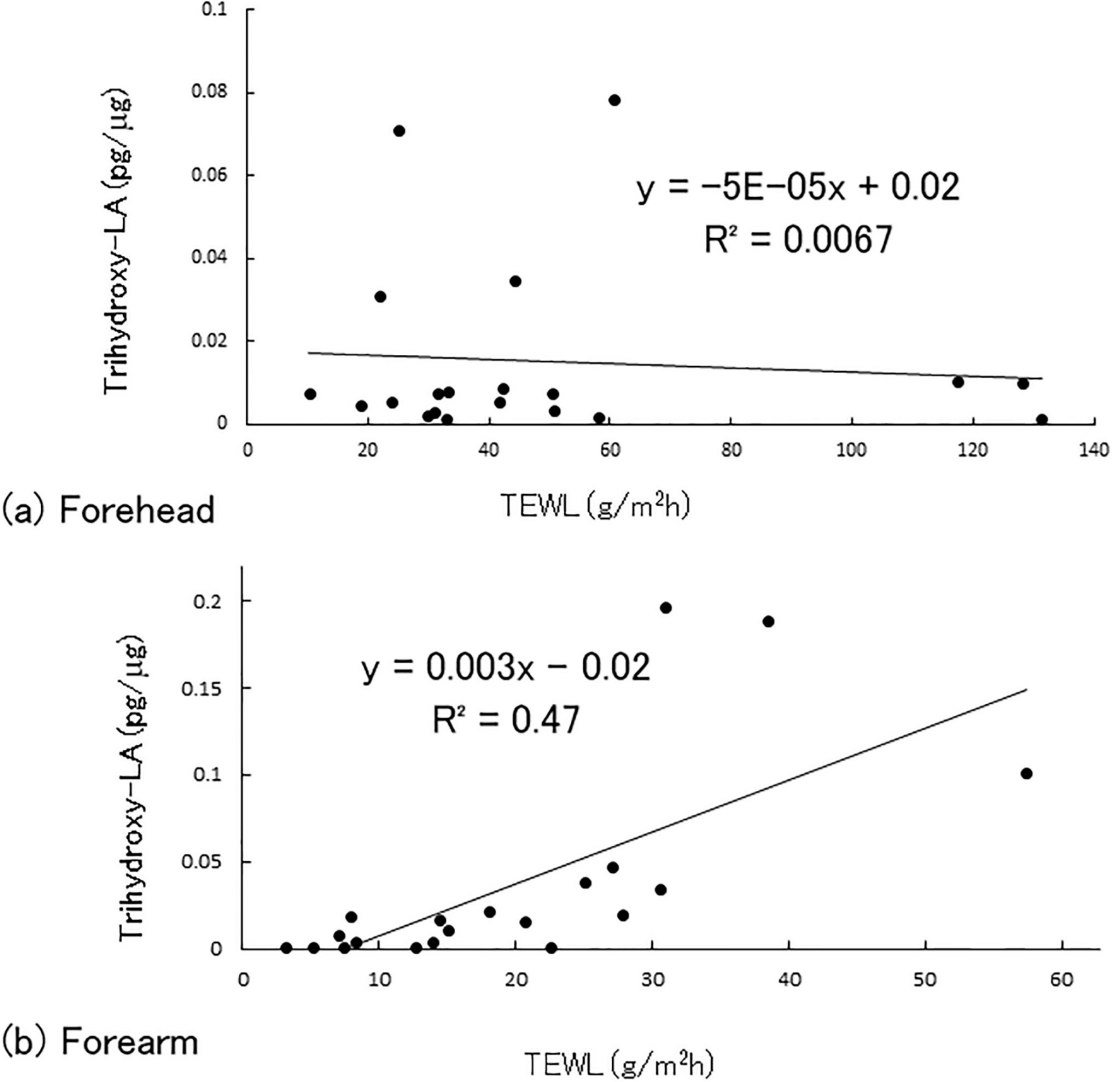
Correlation coefficients between trihydroxy-linoleic acid and TEWL. (a) Forehead skin and (b) forearm skin of non-lesional area in atopic dermatitis.

We confirmed that TEWL and the amount of trihydroxy-linoleic acids were increased in atopic dermatitis patients; therefore, we next examined the correlation between them. In forehead skin, TEWL had no correlation with the amount of trihydroxy-linoleic acids (Fig 3a). On the other hand, TEWL in forearm skin was positively correlated with the amount of trihydroxy-linoleic acids (R^2^ = 0.47, *p* < 0.01; Fig 3b). We also analyzed the correlations of clinical laboratory data such as IgE, peripheral eosinophil count, TARC, LDH, and SCORAD with the amount of trihydroxy-linoleic acids in the atopic dermatitis group, but found no significant results (S4 Table).

### ALOX12B/ALOXE3/ABHD9 expression in NHEK upon IL-4 treatment

**Fig 4:**
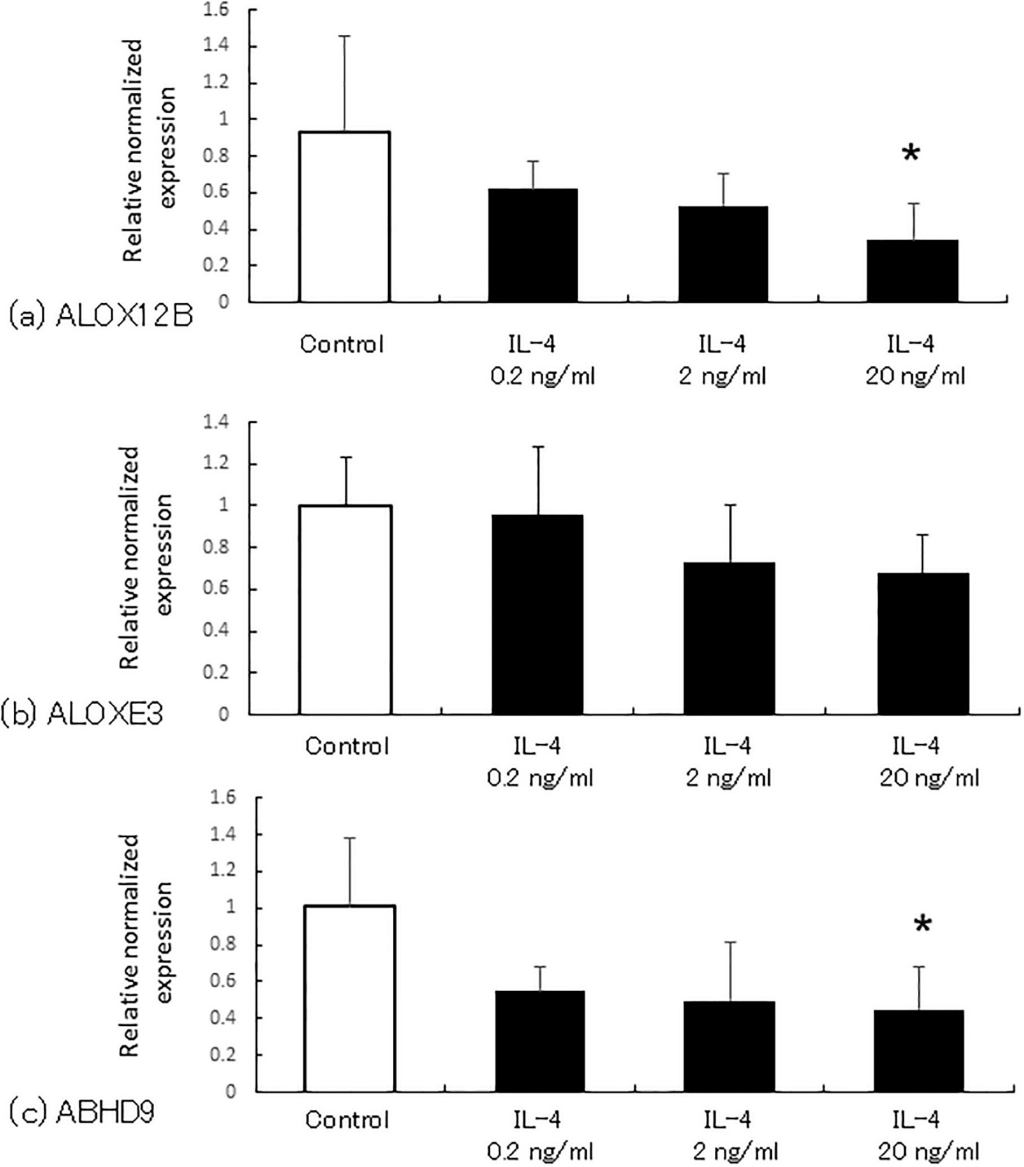
The mRNA expression of enzymes involved in CLE formation upon IL-4 stimulation. ALOX12B, ALOXE3 and ABHD9 mRNA levels of primary normal human epidermal keratinocytes were measured by real-time RT-PCR. The mean values of threshold cycle were 27.5 ± 0.5 (ALOX12B), 22.4 ± 0.1 (ALOXE3), 23.1 ± 0.4 (ABHD9), 15.4 ± 0.4 (β-actin), respectively. The amount of mRNA was normalized to that of β-actin using the ΔCt method, as described in the manufacturer’s protocol. Data are expressed as mean ± SD (n = 6). One-way ANOVA with repeated measures was used for the comparison. Because the initial P value was less than 0.05, Scheffe’s test was used to determine the significance between groups. *p < 0.05 versus control.

We assumed that the increase of trihydroxy-linoleic acids in atopic dermatitis patients is due to the increased gene expression of enzymes involved in CLE formation, ALOX12B (12*R*-LOX), ALOXE3 (eLOX3), and ABHD9 (EPHX3). Hence, these mRNA expressions in NHEK upon stimulation with IL-4, one of the typical inflammatory cytokines in atopic dermatitis, was investigated. As shown in Fig 4a and 4c, ALOX12B and ABHD9 mRNA expression tended to be downregulated by IL-4. IL-4 clearly did not increase the expression of the three enzymes (Fig 4a–4c).

## Discussion

In this study, our results revealed that triols in human stratum corneum can be simply measured by LC-MS using stripped tape specimens. We previously reported that trihydroxy-linoleic acid, triol, esterified to fatty acid was mainly composed of 9*R*,10*R*,13*R*- and 9*R*,10*S*,13*R*-enantiomers (S1 Fig) in human epidermis [16]. Their pure triol chirality is due to metabolism by a series of enzymes, 12*R*-LOX oxidation of linoleate, the product of the eLOX3 transformation of 9*R*-HPODE to epoxyalcohol, and the epoxide hydrolysis of epoxyalcohol by epoxide hydrolase [9, 11]. Then, the trihydroxy-linoleate is cleaved from fatty acid to form ceramide OS (Fig 1b). Compared with the amount of esterified triols, free (non-esterified) triols are present at quite a low level, without specific isomers. It is generally considered that this series of pathways enables ceramide EOS species to be metabolized to produce free ceramide OS for covalent coupling to proteins and construction of the CLE [1, 16].

As shown in Fig 1a, CLE plays an indispensable role in combining the corneocyte envelope with extracellular lamellar lipids. It has been reported that the amount of stratum corneum ceramides in patients with ichthyosis or atopic dermatitis is lower than in normal subjects [19, 21, 28]. This paucity of ceramides in these diseases is involved in the decrease of ceramide OS comprising the CLE [4]. Although there is thus a need to measure the ceramide OS reflecting stratum corneum ceramides, there are a range of obstacles impeding this. For example, ceramide OS have long carbon chains of different lengths in their fatty acid structures. Moreover, in LC-MS system, there was a carryover problem due to adhesion of OS ceramide to LC-column.

For this reason, we measured trihydroxy-linoleic acid as a final metabolite in the process of ceramide OS formation. We predicted that, in atopic patients, trihydroxy-linoleic acid would decrease in parallel with ceramide OS. However, contrary to our expectations, it was detected in the forearm of atopic dermatitis patients at levels nearly 10 times higher than in healthy subjects (protein quantity correction). We thus predicted that the expression of 12*R*-LOX, eLOX3, and EPHX3, which are needed to produce triols, might be elevated in this disease. However, the expression of these mRNAs was not upregulated in NHEK in the presence of the allergic inflammatory cytokine IL-4. One potential reason for the increase of triols is the difference in the amount of substrate, linoleic acid, between healthy subjects and patients with atopic dermatitis. Indeed, linoleic acid concentration in the plasma and adipose tissue of atopic dermatitis patients was significantly elevated [29, 30].

Moreover, in atopic dermatitis patients who have impaired skin barrier function, ceramide levels are altered. Several mechanisms have been suggested to contribute to the decrease in ceramide content in such cases. For example, an increase of kallikrein activity in atopic dermatitis suppresses ceramide-generating enzymes such as acidic sphingomyelinase and β-glucocerebrosidase [31]. In addition, the upregulation of interferon gamma expression in atopic dermatitis downregulates the epidermal synthesis of ceramides [32]. Namely, the percentage and amount of ceramide EOS as a source of triols were decreased in non-lesional skin of atopic dermatitis patients [33].

Taking these findings together, it is considered that the level of ceramide EOS binding to linoleic acid is decreased, but abundant linoleic acids that are not effectively depleted may be metabolized by enzymes (12*R*-LOX/eLOX3/epoxide hydrolase) without binding ceramide EOS. This indicates that the effective upregulation of ceramide EOS synthase activity might be able to normalize the construction or function of the stratum corneum barrier.

Intriguingly, trihydroxy-linoleic acid levels were positively correlated with TEWL of non-lesional forearm skin in patients with atopic dermatitis (R^2^ = 0.47, *p* < 0.01). Although these triols may reflect inefficient metabolization, the biological action of trihydroxy-linoleic acid for skin barrier function is still unknown. An increase of trihydroxy linoleic acid might be possible to downregulate ceramide EOS synthesis. On the other hand, TEWL of non-lesional forehead skin had a low correlation with triols. This may have been because the amount of triols was lower than that of forearm or due to another anatomical difference associated with this site [34]. In healthy subjects, such correlation was not observed in both forehead and forearm skin.

In this study, we were able to simply and practically measure trihydroxy-linoleic acid by a tape-stripping method and showed for the first time that the trihydroxy-linoleic acid levels in the stratum corneum were elevated in patients with atopic dermatitis compared with those in healthy individuals. A significant correlation between trihydroxy-linoleic acid level and TEWL in forearm of atopic dermatitis was identified. Since tape-stripping sampling is non-invasive, trihydroxy-linoleic acid levels may be useful for monitoring barrier function in atopic dermatitis.

## Supporting information

S1 Fig Eight isomers of 9,10,13-trihydroxy-11*E*-octadecenoic acid. In human skin, 9*R*,10*R*,13*R*- (triol-1) and 9*R*,10*S*,13*R*- (triol-3) trihydroxy-11*E*-octadecenoate account for over 95% of the enantiomers of trihydroxy-linoleic acid [16].
(TIF)

S1 Table Clinical and laboratory data of atopic dermatitis patients. (DOCX)

S2 Table Primers for quantitative polymerase chain reaction. (DOCX)

S3 Table Correlation between SCORAD and clinical laboratory data. (DOCX)

S4 Table Coefficient of determination (R^2^) and *p*-value between trihydroxy-linoleic acid and various clinical and laboratory parameters in atopic dermatitis patients. IgE, immunoglobulin E; TARC, thymus and activation-regulated chemokine; LDH, lactate dehydrogenase; SCORAD, SCORing Atopic Dermatitis.
(DOCX)
