# Supplementary figures and images for: Measurement of trihydroxy-linoleic acids in stratum corneum by tape-stripping: Possible biomarker of barrier function in atopic dermatitis
Source: PLoS One. 2019 Jan 4;14(1):e0210013. doi: 10.1371/journal.pone.0210013 (PMC6319710; doi:10.1371/journal.pone.0210013)

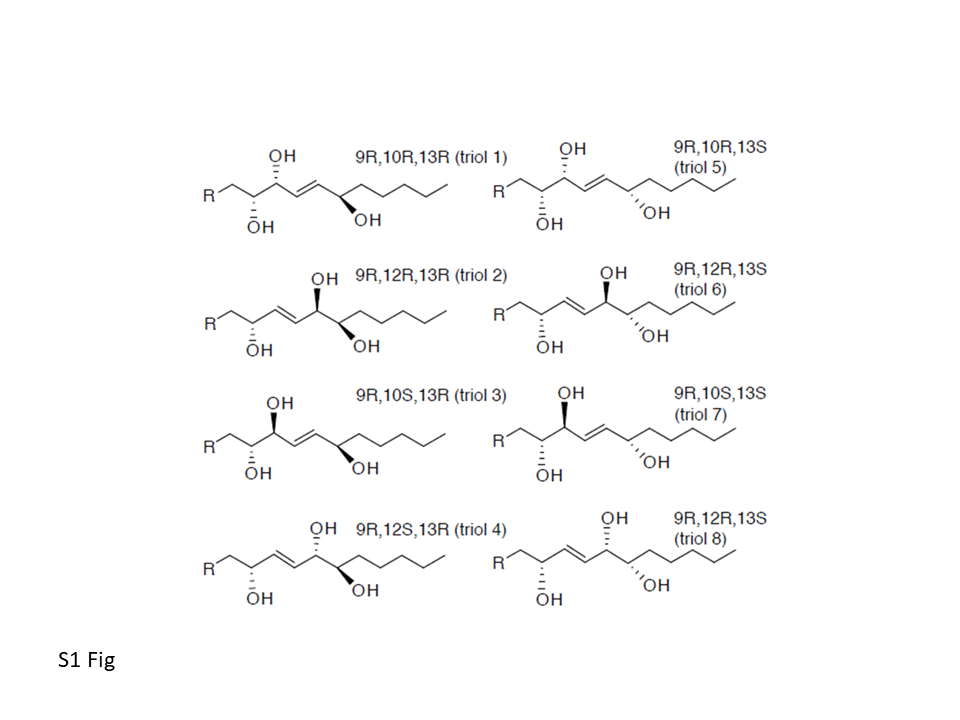

Supplement: S1 Fig — In human skin, 9R,10R,13R- (triol-1) and 9R,10S,13R- (triol-3) trihydroxy-11E-octadecenoate account for over 95% of the enantiomers of trihydroxy-linoleic acid [16]. (TIF) [file pone.0210013.s001.TIF]
