# Supplementary material for: Measurement of trihydroxy-linoleic acids in stratum corneum by tape-stripping: Possible biomarker of barrier function in atopic dermatitis
Source: PLoS One. 2019 Jan 4;14(1):e0210013. doi: 10.1371/journal.pone.0210013 (PMC6319710; doi:10.1371/journal.pone.0210013)
Supplement: S1 Table — (DOCX) [file pone.0210013.s002.docx]

Clinical and laboratory data in atopic dermatitis patients

| Parameter |  |
| --- | --- |
| Patient number | 20 |
| Age (years, mean ± SD) | 42.6 ±13.8 |
| Sex (M:E; %) | 55 : 45 |
| Serum-IgE (IU/ml, mean ± SD) | 10,662.0 ±15,793.1 |
| Eosinophils (%, mean ± SD) | 9.86 ±6.90 |
| TARC (pg/ml, mean ± SD) | 3040.9 ±669.2 |
| LDH (U/l, mean ± SD) | 255.5 ±21.9 |
| SCORAD (mean ± SD) | 35.8 ±4.6 |
| Treatment |  |
| Emollient (number) | 10 |
| Tacrolimus (number) | 3 |
| Topical steroid (number) | 13 |
| Anti-histamine (number) | 5 |
| Cyclosporine (number) | 2 |

The normal ranges are as follows: serum-IgE, 5-160 IU/ml; eosinophils, 1%-8%;

TARC, <450 pg/ml; LDH, 115-208 U/l

S1 Table
