# Supplementary material for: Measurement of trihydroxy-linoleic acids in stratum corneum by tape-stripping: Possible biomarker of barrier function in atopic dermatitis
Source: PLoS One. 2019 Jan 4;14(1):e0210013. doi: 10.1371/journal.pone.0210013 (PMC6319710; doi:10.1371/journal.pone.0210013)
Supplement: S2 Table — (DOCX) [file pone.0210013.s003.docx]

Primers for quantitative polymerase chain reaction

| Gene | Forward primer | Reverse primer |
| --- | --- | --- |
| ALOX12B | 5’-CAAGCAGGACTTCTACCACTG-3’ | 5’-GAGGGCGGTAACTGGGAAT-3’ |
| ALOXE3 | 5’-TGTCACCGAACCGGATGGTA-3’ | 5’-CGGTAGCATTCTTGTCGGG-3’ |
| ABHD9 | 5’-TGTGGACTTGCGAGGCTATG-3’ | 5’-ATGCACTTCGAGTAACCCAGG-3’ |
| β-actin | 5’-ATTGCCGACAGGATGCAGA-3’ | 5’-ATTGCCGACAGGATGCAGA-3’ |

S2 Table
