# Supplementary material for: Measurement of trihydroxy-linoleic acids in stratum corneum by tape-stripping: Possible biomarker of barrier function in atopic dermatitis
Source: PLoS One. 2019 Jan 4;14(1):e0210013. doi: 10.1371/journal.pone.0210013 (PMC6319710; doi:10.1371/journal.pone.0210013)
Supplement: S3 Table — (DOCX) [file pone.0210013.s004.docx]

SCORAD

| Parameter | R^2^ | *p* value |
| --- | --- | --- |
| Serum-IgE (IU/ml) | 0.22 | 0.02 |
| Eosinophil (%) | 0.32 | <0.01 |
| TARC (pg/ml) | 0.57 | <0.01 |
| LDH (U/l) | 0.57 | <0.01 |
| TEWL forehead (g/m^2^h) | 0.01 | 0.88 |
| TEWL forearm (g/m^2^h) | 0.04 | 0.38 |

S3 table
