# Supplementary material for: Measurement of trihydroxy-linoleic acids in stratum corneum by tape-stripping: Possible biomarker of barrier function in atopic dermatitis
Source: PLoS One. 2019 Jan 4;14(1):e0210013. doi: 10.1371/journal.pone.0210013 (PMC6319710; doi:10.1371/journal.pone.0210013)
Supplement: S4 Table — IgE, immunoglobulin E; TARC, thymus and activation-regulated chemokine; LDH, lactate dehydrogenase; SCORAD, SCORing Atopic Dermatitis. (DOCX) [file pone.0210013.s005.docx]

SCORAD

|  | Trihydroxy-linoleic acids  forehead | | Trihydroxy-linoleic acids  forearm | |
| --- | --- | --- | --- | --- |
| Parameter | R^2^ | *p* value | R^2^ | *p* value |
| Serum-IgE (IU/ml) | 0.01 | 0.80 | 0.03 | 0.49 |
| Eosinophils (%) | 0.12 | 0.14 | 0.09 | 0.20 |
| TARC (pg/ml) | 0.01 | 0.69 | 0.05 | 0.36 |
| LDH (U/l) | 0.10 | 0.17 | 0.13 | 0.12 |
| SCORAD | 0.02 | 0.58 | 0.08 | 0.22 |

S4 table
